# Assessing Mediterranean Diet Adherence with the Smartphone: The Medipiatto Project

**DOI:** 10.3390/nu12123763

**Published:** 2020-12-07

**Authors:** Maria F. Vasiloglou, Ya Lu, Thomai Stathopoulou, Ioannis Papathanail, David Faeh, Arindam Ghosh, Manuel Baumann, Stavroula Mougiakakou

**Affiliations:** 1 ARTORG Center for Biomedical Engineering Research, University of Bern, 3008 Bern, Switzerland; maria.vasiloglou@artorg.unibe.ch (M.F.V.); ya.lu@artorg.unibe.ch (Y.L.); thomai.stathopoulou@artorg.unibe.ch (T.S.); ioannis.papathanail@artorg.unibe.ch (I.P.); 2 Epidemiology, Biostatistics and Prevention Institute (EBPI), University of Zurich, 8001 Zurich, Switzerland; david.faeh@uzh.ch; 3 Oviva S.A., 8852 Altendorf, Switzerland; arindam.ghosh@oviva.com (A.G.); manuel.baumann@oviva.com (M.B.)

**Keywords:** Mediterranean diet, Mediterranean diet score, Mediterranean diet adherence, artificial intelligence, machine learning, smartphone, computer vision

## Abstract

The Mediterranean diet (MD) is regarded as a healthy eating pattern with beneficial effects both for the decrease of the risk for non-communicable diseases and also for body weight reduction. In the current manuscript, we propose an automated smartphone application which monitors and evaluates the user’s adherence to MD using images of the food and drinks that they consume. We define a set of rules for automatic adherence estimation, which focuses on the main MD food groups. We use a combination of a convolutional neural network (CNN) and a graph convolutional network to detect the types of foods and quantities from the users’ food images and the defined set of rules to evaluate the adherence to MD. Our experiments show that our system outperforms a basic CNN in terms of recognizing food items and estimating quantity and yields comparable results as experienced dietitians when it comes to overall MD adherence estimation. As the system is novel, these results are promising; however, there is room for improvement of the accuracy by gathering and training with more data and certain refinements can be performed such as re-defining the set of rules to also be able to be used for sub-groups of MD (e.g., vegetarian type of MD).

## 1. Introduction

The Mediterranean diet (MD) is characterized by a high intake of vegetables, fruits, nuts, legumes, complex carbohydrates, unsaturated lipids (mainly olive oil), a moderate consumption of fish and alcohol, and a low intake of red meat [1,2,3]. MD best fulfils the criteria of what is regarded as a healthy eating pattern and has also been shown to decrease the risk of various non-communicable diseases, such as type 2 diabetes, cardiovascular disease, and cancer [4,5,6,7,8]. It has also been shown to be effective in sustainably lowering body weight [9]. MD is a food pattern because it represents a set of foods and nutrients that work as a whole, with a positive impact on human health that repeats over time [10]. Optimal adherence to the MD can be visualized in the form of a pyramid [11]: at the base, there are foods that should be consumed frequently and at the upper levels, foods that are advised to be eaten in small to moderate amounts. In fact, a healthy or unhealthy diet relies on the quantity and the frequency of the consumed food. The pyramid illustrates guidelines recommending food intake based on a meal, a daily- or a weekly-based consumption.

Mediterranean diet adherence (MDA) is a score-based evaluation metric used to assess how closely individuals follow the MD, i.e., a higher MDA score indicates close adherence to MD. The MDA is calculated based on each single meal within a long-term diet (daily and weekly) [12]. To the best of our knowledge, there is no efficient approach, manual or automatic, able to perform automatic MDA assessment, since no such tool exists, but also because there is no agreed upon set of rules for this type of assessment among dietitians and healthcare professionals. This makes it difficult for people to follow MD and hinders the dissemination of such a healthy food pattern. However, in the last decade, artificial intelligence (AI) techniques [13,14,15,16,17,18,19] have opened new possibilities for automatic dietary assessment by directly analyzing food images. Results from a global survey conducted among healthcare professionals (HCP) (*n* = 1001), found out that HCP would like to recommend to their patients/clients apps that are easy to use, validated, and automatically produce nutrient and calorie estimations [20].

In contrast to typical dietary assessment systems [18,21,22] that demand an accurate food volume estimation, the MDA assessment requires a rough estimation of the food serving size, but rather a fine food recognition. To meet this requirement, it is fitting to perform accurate multi-label food recognition from a single image, to precisely recognize multiple food types even when they are mixed together.

**Figure 1:**
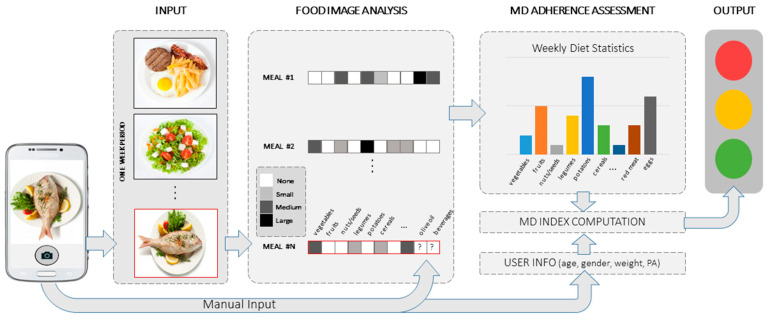
Outline of the medipiatto system.

It has been described that electronic diaries which depict food items might improve adherence to self-monitoring [23]. Thus, in this paper, we propose an AI-based smartphone application (app) to automatically assess the user’s MDA. Figure 1 shows the outline of the proposed system. The input to the app is a single RGB (Red, Green, Blue) food image captured by the user. The app analyzes the image to recognize the different foods and portions. The analysis regards certain food categories and their portions, based on a set of rules defining MDA, which are formulated in the current manuscript. On top of the automatically recognized foods, the user needs to manually add certain non-visually detectable food types or ingredients (e.g., olive oil, salt, etc.) that are of importance for the MDA estimation. This step is unavoidable even with manual estimation performed by experienced dietitians since certain ingredients that are mixed into the food or in the sauce are only known to the persons that prepared the food. The analysis of each image is stored by the app and used at a later stage, as there are certain food categories that affect MDA on a per-meal, daily, or weekly basis. At the end of each week, all analyses are processed, and the MDA score is estimated. This score is presented to the user.

The core algorithm used in the image processing module is a graph convolutional network (GCN) [24,25,26] for multiple food type recognition and serving size estimation. The input of the algorithm is a single view RGB image, while the outputs are all the food categories contained in the image and their corresponding serving sizes. This approach incorporates food category semantics into the learning process and is able to explore the relation among different food categories, so as to achieve better recognition accuracy, especially for cases of overlapping foods. To estimate the rough food serving size, a regression layer is designed and integrated into the GCN framework, which outputs the serving size of each food category.

Comparing to existing food recognition and volume estimation approaches, which either recognize only one food category per image [27] or address the recognition of different food categories separately [28], the advantages of the GCN-based proposed algorithm include: (1) the user only needs to capture one photo including all the food items under evaluation, which significantly reduces the burden of image capturing; (2) the proposed algorithm has the ability to take advantage of the correlations between certain food categories in the benefit of accuracy for the cases where the food items are mixed together. It should be noted that, although the simple regression for the serving size estimation would not be as accurate as the food volume estimation of other dietary assessment systems, which utilize 3D information (from multi-view or depth sensor), the accuracy of food serving size estimation is adequate for MDA assessment. In addition, as a trade-off solution, the approach for serving size estimation allows for better performance of the food recognition module.

The contributions of this paper include:

(1) The introduction of a concrete set of rules regarding the assessment of MDA. This is implemented based on the MD score that we use in the current manuscript. The rules that are introduced take into account the type, frequency, and quantity of the consumed foods and refer to the scoring system chosen for this manuscript.
(2) The design and development of an innovative AI-based system for automatic MDA assessment, which estimates the user’s long- and short-term adherence to MD. To the best of our knowledge, the proposed system is the first AI-based system for MDA assessment. The designed core algorithm embedded in the proposed system is able to recognize multiple food items and their serving sizes simultaneously from a single food image. The experimental results demonstrate the better performance of the designed algorithm compared to the widely used baseline algorithm.
(3) A comparison study between the proposed system and four experienced dietitians for MDA assessment on the food images captured under free-living condition was conducted. The results of the study indicate similar results between the proposed system and the experienced dietitians.

### 1.1. Related Work

#### 1.1.1. MDA Evaluation

It is essential to determine the MD adherence level using accurate measurement tools and it has been shown that dietary scores are useful since they depict the consistency of food consumption to a pattern and in compliance to the recommended intake [29]. Dietary scores combine foods and/or nutrient constructs aiming at estimating overall dietary quality and its association to health outcomes [30]. There are many different MD scores tailored to different populations and age groups. The first one was published by Trichopoulou et al. [10] and assessed the adherence to traditional MD. With regards to content validity, the majority of scores are based on negative and positive components, even though there is no consensus on the meaning of the ratings [30], by scoring positively beneficial foods that are mostly consumed in traditional MDs and negatively foods which are less frequently consumed and which are non-typically Mediterranean [31]. Thus, a high score indicates good adherence to the MD and a low score, poor adherence [30]. However, the methodology behind the assessment of MDA scoring is not universally agreed-upon, because of the absence of agreement regarding the definition of MD itself. Since there are different indices with significant variations, there is a need for a clarification in terms of the number of components, the contribution of each of them in the indices and the scoring criteria in order to improve the reliability and agreement between them [32].

#### 1.1.2. AI in Dietary Assessment

The first two steps of automatic dietary assessment are usually treated by directly employing well-established segmentation [33,34] and recognition algorithms [13,17,33,35,36], which can be readily realized using one RGB-image input. With the rapid development of convolutional neural networks (CNNs), the performance of these two steps [33,37,38] can nowadays significantly outperform the traditional approaches that are based on hand-crafted features [34,35].

However, there is not much research focused on food volume estimation. The implementation of traditional volume estimation techniques [38,39,40] for food requires more than one food image, either as a video sequence [41,42], or as two individual input images [14]. A more robust 3D food model reconstruction can be achieved by processing an RGB-image and its corresponding depth image captured by a depth sensor [22,43,44]. These approaches achieve high accuracy; however, their implementation remains inconvenient as the depth sensor is not universally available for all end-users. The performance of these approaches, though, is inevitably compromised by the almost always unknown information of the non-visible parts of the food.

As a simpler alternative, supervised CNNs permit the use of a single RGB-image as input [15,43,45] for the prediction of the corresponding depth map needed for 3D food model building [15,43] or the direct food volume regression [45].

## 2. Methodology

### 2.1. Data Gathering

For the development of the app and training of the algorithms, a large number of appropriately annotated data is needed. This data refers to images depicting food, upon which the GCN is trained, so as to recognize the food groups that the users consume and estimate the corresponding servings. These images need to be annotated in terms of food categories and respective serving sizes. A group of annotators have undertaken this task and needed to be aware of the foods that are of interest and their corresponding serving size. A questionnaire has therefore been created, providing basic instructions for the annotation process. It lists the food groups of interest, their respective serving sizes, and examples of how the annotator can recognize and correctly annotate each food.

### 2.2. Annotators’ Questionnaire

This questionnaire was created based on MD adherence questionnaires [46] aided by the unit sizing, in order to support the annotators in the food estimations. We chose to mostly include unit sizing of household measures (e.g., cups) and hand references (e.g., handful), as it was deemed most appropriate and easiest for people with no experience in dietetics to follow. There were still certain issues, however, that needed to be considered. First, the serving sizes vary by food and differ in some foods depending on whether they are dry or in edible form. Furthermore, each country has different serving sizes and, in many cases, there is no nationwide consensus and/or the precise units are not publicly available. For these reasons, we decided to use the available and consensus-based serving sizes as published by the British Nutrition Foundation [41]. However, since it does not sufficiently cover all food groups (e.g., dairy), if the serving size for a food item was not available, we used serving sizes as published by the Swiss Food Association [44]. The precise definition of serving sizes, as well as certain other specifying instructions, e.g., only raw or cooked vegetables are considered, while vegetable juice is ignored, helped to avoid/reduce the confusion of annotators.

For food categories that are of importance for MD, but undetectable by the app, e.g., oil, melted butter, etc. and for beverages that may easily be misclassified e.g., water vs. vodka, the user will need to either manually add the appropriate information or verify/correct any incorrect recognition.

### 2.3. Rules’ Definition for MD Scoring

A set of rules has been defined, which translates the composition of MD compatible food categories, consumed servings, and frequency on a weekly basis, into an MD adherence index, which can be then associated with three output levels: low, medium, and high adherence. The analysis was based on already established scoring methodologies [47]. The resulting set of rules is shown in Table 1.

Depending on the consumption of MD food items, an MD score will be provided to the user on a weekly basis. The score lies within the range of 0 (no adherence) to 24 (highest adherence). It should be noted that not all food items that are assessed are used for the calculation of the classical MD adherence. The information on non-white pasta and rice is used for a future updated version of MD adherence.

To our knowledge, there is no concrete scientific consensus regarding the detailed scoring procedure that should be used, other than the given maximum scores. The procedure we use is the following:

#### 2.3.1. Components That Are Counted Daily or per Meal

Meal-based categories (e.g., fruits) refer to the three main meals of the day, namely breakfast, lunch, and dinner. Each meal that contains at least one category contributes one point. More than one serving of a category or more than one categories contained in a meal do not contribute extra points.

These points are then summed up on a daily basis, contributing to the overall score by a maximum of 3 points.

Food groups that are counted on a daily basis, contribute points any time that they are consumed, regardless of the meal or time of day. Their servings are counted throughout the day and at the end they contribute their corresponding points. Nuts contribute 2 points, when one or more servings are consumed. Dairy products contribute one point for one serving and 2 points for two or more servings per day. Finally, fermented beverages contribute one point for 1 or 2 servings per day and 0 points for 3 or more servings per day.

After one week, the points gathered the meal- and daily-based food groups are summed up and divided by 7 in order to receive the daily average of these two categories.

#### 2.3.2. Components That Are Counted Weekly

For the scoring of food groups that are counted on a weekly basis, servings are summed up for the entire week, consequently contributing their corresponding points. Legumes, eggs, fish, and white meat contribute one point if there are 2 or more servings per week. Red meat contributes 1 point if there is a maximum of one serving of it per week and 0 points if there are more. Finally, sweets contribute one point if there are one or two servings per week and 0 points if there are more. More detailed tables regarding the different food groups’ scoring can be found in Appendix A.

#### 2.3.3. Total Score Calculation

Weekly MD adherence = ((sum of meal scoring + daily scoring)/7) + weekly scoring(1)

After the initial assessment that has to last one week, the meal- and daily-based adherence to the MD will be used to calculate and display a preliminary adherence trend, based on Equation (1). However, a definite new score and classification can only be generated after the inclusion of the next entire week-based analysis.

#### 2.3.4. Food Recognition and Serving Size Estimation

**Figure 2:**
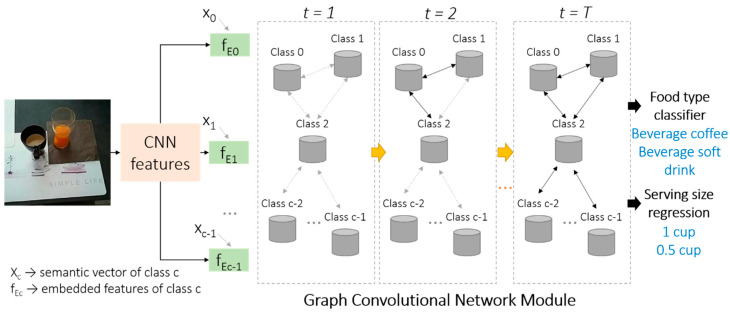
Overall framework of graph representation learning for multi-label food recognition and serving size prediction. *c* is the index of the food category; *t* indicates the iterative process for the graph convolutional network module training.

The graph representation learning method [24,25,26] is designed for food recognition and serving size estimation. The overall network architecture is shown in Figure 2.

A CNN (here we use the ResNet101 [49]) is initially applied on the entire input RGB food image for feature extraction. The extracted image features are then embedded with the word semantic features for each food category. The word semantic features are extracted using the pre-trained GloVe [50] model. The feature embedding strategy used in this project is illustrated in Figure 2 and described by Equations (2)–(4), which are based on a semantic guided attention mechanism [25] and widely used for cross-modal feature fusion purpose in the literature [25,26].

(2) $$f_{Ec}=\underset{i}{\sum }atten_{c,i}f_{c,i}$$

(3) $$f_{c,i}=P^{T}\left(tanh\left(\left(U^{T}f_{img,i}\right)⊙\left(V^{T}f_{word}\right)\right)\right)+b$$

(4) $$atten_{c,i}=softmax\left(F_{a}\left(f_{c,i}\right)\right)$$

where c is the index of the food category and *i* indicates the pixel location within the image $f_{Ec}$ are the embedded features for category c and $f_{img}$ represents the extracted image features, while the $f_{word}$ are the word semantic features. The P, U, V and b are the parameters to be learned. ⊙ is the element-wise multiplication operation. $F_{a}$ is the attentional function that is implemented as a fully connected layer.

After obtaining the embedded features corresponding to all the food categories, a graph G={V,A} is introduced to build the GCN [26], where the nodes $V\to \left\{v_{0},v_{1},\ldots ,v_{C-1}\right\}$ refer to the food categories and the edges $A\to \left\{a_{0,0},a_{0,1,}\ldots ,a_{0,C-1},\ldots ,a_{C-1,C-1}\right\}$ indicate the co-occurrence of the corresponding categories, in which C is the total image categories contained in the database. *V* is represented using the embedded features and the A is computed using the label annotations of the samples of the training set. After several iterations of the process executed by the GCN module [24,25], the network will produce the correlated features for each food category. The detailed iteration strategies are similar to those in [25]. The extracted correlated features are then concatenated and provided to the food classification and serving size recognition layers. These two layers are implemented as fully connected and output the food recognition results and the corresponding serving size, respectively.

During network training, the input image is resized to 512 × 512. The binary cross entropy loss is applied to the food classification layer, while the mean relative squared error is used for the serving size regression layer. The Adam optimizer with learning rate 1 × 10^−5^ is applied during the network training. The batch size is set at 32 and the number of iterative steps of the GCN module is set at 3.

## 3. Results

### 3.1. Database

The images analyzed were captured by the users of the Oviva [51] platform, which agreed on using these images for this purpose, under free-living conditions and contains 5776 food images. There were no inclusion or exclusion criteria regarding the users or the images and the data is fully anonymized. Each image was annotated by 5 different inexperienced annotators regarding the food type and serving size contained, selecting from a total of 31 food categories (Table 2) defined by the experienced dietitians that have determined the MDA ruleset. We split the database into a training and a testing set, which includes 5483 and 293 images, respectively.

The five annotations of each image are then averaged in both sets and set as the ground truth. For the testing set, we additionally involved an experienced dietitian to correct the average annotations of the inexperienced annotators, to ensure the correctness of the testing ground truth and the fairness of the evaluation results.

**Figure 3:**
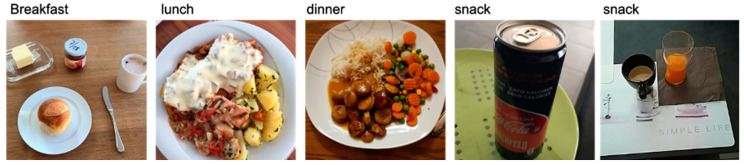
Example daily sub-set.

For the MDA assessment, we randomly select 71 images from the testing set and divide them into 14 sub-sets, each sub-set containing 3–7 food images, representing the daily intake of one user for one day, resulting in daily intake information of one user for two consecutive weeks. We manually marked all the testing images as “breakfast,” “lunch,” “dinner,” or “snack” based on common sense with respect to the different meal types. Figure 3 shows an example of one daily sub-set.

### 3.2. Food Recognition and Serving Size Estimation

The mean Average Precision (mAP) metric is used to evaluate the performance of the multi-label food recognition. mAP is the most commonly used evaluation metric for multi-label image classification in literature [24,25]. The calculation of mAP is represented in Equation (5).

(5) $$mAP=\frac{1}{C}\underset{c}{\sum }mean\left(max\left(P_{R^{c}}^{c}\right)\right)$$

where: *C* indicates the number of food categories, while c is its index, *P* indicates the precision: P=truepositive/(truepositive+falsepositive), *R* indicates the recall: R=truepositive/(truepositive+falsenegative) and $max\left(P_{R^{c}}^{c}\right)$ indicates the max precision for each recall level (value) of category c.

To evaluate the performance of serving size estimation, the mean Absolute Percentage Error (mAPE) is applied as the evaluation metric. mAPE is described by Equation (6).

(6) $$MAPE=\frac{1}{N_{itm}}\underset{itm}{\sum }\frac{\left|S_{gt}^{itm}-S_{pred}^{itm}\right|}{S_{gt}^{itm}}$$

where: *itm* is the index of the correctly predicted food items of the testing images, $S_{gt}^{itm}$ and $S_{pred}^{itm}$ are the ground truth and predicted serving size of the corresponding food item, respectively and $N_{item}$ is the number of evaluated food items.

**Figure 4:**
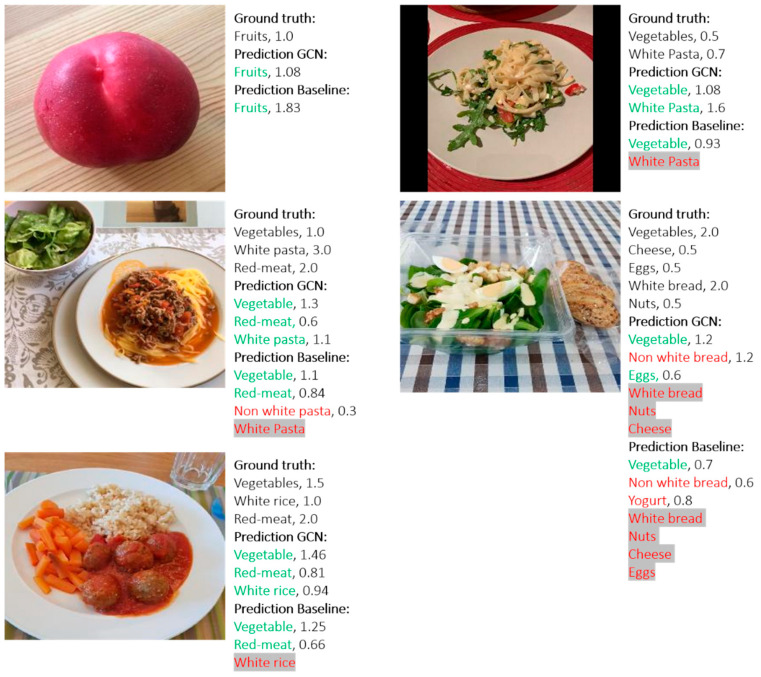
Example results of the GCN-based algorithm. Green indicates correct prediction and red indicates incorrect prediction. Red with gray background indicates missing prediction.

Table 3 shows the comparison results of the developed approaches with respect to the multi-label food recognition and serving size estimation. To demonstrate the good performance of the developed approaches, we implemented the widely used ResNet101 [49] as a baseline method. To implement the baseline, a dense layer with sigmoid activation function is connected to the last average pooling layer of ResNet101 for multi-label food recognition. In addition, another dense layer is implemented in parallel and connected to the average pooling layer for the serving size estimation of each predicted food category. During implementation, we apply the pre-trained weight from ImageNet to initialize the backbone ResNet101, for a fair comparison with the proposed GCN-based method. As seen in Table 3, the GCN-based method achieves better performance in both multi-label food recognition and serving size estimation, which exceeds the baseline method by 11% and 2% for food recognition and serving size estimation, respectively. The experiments are conducted on a server equipped with GTX1080ti GPUs and the computation time of the different methods for a single food image is listed in Table 3. Figure 4 illustrates some example results of the proposed method.

### 3.3. MD Adherence Assessment

The MDA scores of the developed system are calculated based on the food recognition and serving size estimation from the algorithm and the scoring rules presented in the current manuscript. For this experimental phase, we used the 71 images that have been divided into 14 sub-sets representing the daily intake information of one user for two consecutive weeks, as described in Section 3.1. Four experienced dietitians manually calculated the MDA score for these sub-sets. One of the experienced dietitians has been involved in the creation and correction of the used database. All four were asked to output a weekly MDA score based on the scoring system that has been developed.

Table 4 shows the comparison results of the proposed system and the estimation of the experienced dietitians. “System-I” indicates the results using the image-level method. It is obvious by these results that the proposed system predicts MDA scores, which are very close to those predicted by the experienced dietitians.

## 4. Conclusions

In this paper, we present a novel system that can be used to monitor the user’s diet, quantify their intake, and assess their adherence to MD. To achieve this, we have developed a set of rules, based on already established scoring methodologies that translate the composition of MD compatible food groups, serving sizes, and frequency on a weekly basis into an MDA score. The final system will be able to translate this MDA score into three MD adherence levels, namely: low, medium, and high, which will be provided to the user in the form of a traffic light system (Table 5). We also designed and developed an algorithm for multi-label food recognition and serving size estimation from single-color images. The experimental results demonstrate that the proposed GCN-based algorithm surpasses the baseline approach in both food recognition and serving size estimation tasks. We also compared the MDA scores calculated by the developed system to the MDA scores estimated by four experienced dietitians and the results show that similar values are produced by both, demonstrating satisfactory performance of our system.

In further developing the system and in order for it to be established as a dietary support, there are still certain issues that need to be addressed and certain aspects that need refinement and improvement. The proposed scoring system can be further refined by not only considering the minimum portions that are consumed, but also consider the cases where the user exceeds the recommended for the MD portions. In addition, a parallel scoring system can be developed and incorporated to the system, which does not only follow the traditional MD, but also other forms. At the moment if a user follows a vegetarian type of MD, they would get a low MDA score, even though the proper nutrients may have been consumed.

Moreover, the current pipeline will inevitably always include the manual input by the user, regarding the undetectable foods (e.g., butter) and their portions. Though usually these foods are consumed in low amounts, this requirement entails a level of uncertainty when it comes to the user and their familiarity with portion estimation or knowledge of the food’s ingredients. The AI modules can be further improved, in the sense of increased accuracy with respect to food recognition and portion size estimation, by optimizing the used algorithms and including more relevant food images in the training process. Thus, the system’s accuracy is expected to increase too. The system’s optimization can be implemented in parallel to user satisfaction and usability assessment, thus improving the overall system, not only on its accuracy but also on its user friendliness.

## Figures and Tables

**Table 1:** Mediterranean diet (MD) Serving Scores.

Mediterranean Diet Serving Score (MDSS)			
Food Category	Recommendation *	Max. Score	Size per Serving ***
Meal basis			
Fruit	1–2 servings or more/main meal **	3	80 g
Vegetables	≥2 servings/main meal **	3	80 g
Cereals ^a^	1–2 servings or more/main meal **	3	50 g
Olive Oil ^b^	1 serving or more/main meal **	3	10 g, 15 mL
Maximum Score:		12	
Daily basis			
Nuts	1–2 servings or more/day	2	20–30 g
Dairy products ^c^	2 servings or more/day	2	
Milk			200 mL
Yoghurt			120–150 g
Cheese			30 g (for soft cheese: 60 g, for cottage cheese: 100 g)
Fermented beverages ^g^	1–2 glass/day (0 points for ≥ 2 glasses)	1	
Wine			100 mL
Beer			250 mL
Maximum Score:		5	
Weekly basis			
Potatoes	≤3 servings/week	1	200 g
Legumes	≥2 servings/week	1	120 g
Eggs	2–4 servings/week	1	120 g
Fish	≥2 servings/week	1	120 g
White meat ^d^	2 servings/week	1	120 g
Red meat ^e^	<2 servings/week	1	120 g
Sweets ^f^	≤2 servings/week	1	20 g
Ice-cream			75 g
Chocolate			30 g
Cookie/pastry			30 g
Sweetened Soft drink			330 mL
Maximum Score:		7	
Total maximum score		24	

* According with the new Mediterranean Diet Pyramid [11]. ** Main meals: breakfast, lunch and dinner. *** Approximately per serving. [27,48]. ^a^ Bread, breakfast cereals, rice and pasta. ^b^ Olive oil used on salads or bread or for frying. ^c^ Milk, yoghurt, cheese, ice cream. ^d^ Poultry, rabbit. ^e^ Pork, beef, or lamb. ^f^ Sugar, candies, pastries, sweetened fruit juices, and soft drinks. ^g^ Wine and beer.

**Table 2:** Food and beverage categories.

Food		Drink
1. vegetables	13. potatoes non-fried	24. beverage—water
2. fruits	14. French fries	25. beverage—coffee
3. nuts	15. meat white	26. beverage—tea
4. legumes/pulses	16. meat red	27. beverage—wine
5. bread white	17. fish/seafood	28. beverage—beer
6. bread non-white	18. eggs	29. beverage—alcoholic *
7. pasta white	19. breaded food	30. beverage—soft-drink
8. pasta non-white	20. sweets	31. beverage—milky coffee
9. rice white	21. milk	
10. rice non-white	22. yogurt	
11. cereals unprocessed	23. cheese	
12. cereals processed		

* alcoholic beverage other than beer or wine.

**Table 3:** Comparison of results for different approaches.

Method	mAP	MAPE	Time (s)
ResNet-101	0.47	63%	0.15
GCN-based	0.58	61%	0.2
	Higher is better	Lower is better	

**Table 4:** Comparison results of the proposed system and the experienced dietitians on MDA scoring.

Time Period	Proposed System	Experienced Dietitians
Week 1	8.4	9.6
Week 2	8.9	8.8

**Table 5:** Levels of MD adherence scores.

MD Score	
0–8	LOW compliance with MD—RED colour
9–15	MEDIUM compliance with MD—ORANGE colour
16–24	HIGH compliance with MD—GREEN colour

## Appendix A

Table A1, Table A2 and Table A3 offer an analytical view of the scoring of the different food groups, which are counted on a meal-, daily- and weekly-basis respectively.

**Table A1:** Scoring of food groups counted on a meal basis

Food Category	Meal	Servings per Meal	Point
Fruits Vegetables	Breakfast	0	0
Cereals Olive oil		≥1	1
Fruits Vegetables	Lunch	0	0
Cereals Olive oil		≥1	1
Fruit Vegetables	Dinner	0	0
Cereals Olive oil		≥1	1

**Table A2:** Scoring of food groups counted on a daily basis

Food Category	Meals	Servings per Day	Points
Nuts	Any	0	0
		≥1	2
Dairy	Any	0	0
		1	1
		≥2	2
Fermented Beverages	Any	0	0
		1–2	1
		>2	0

**Table A3:** Scoring of food groups counted on a weekly basis.

Food Categories	Meals	Servings per Week	Points
Legumes	Any	<2	0
		≥2	1
Eggs	Any	<2	0
		≥2	1
Fish	Any	<2	0
		≥2	1
White Meat	Any	<2	0
		≥2	1
Red Meat	Any	≥2	0
		<2	1
Sweets	Any	>2	0
		≤2	1
